# Alterations in ectopic myocardial contraction assessed using real-time MRI

**DOI:** 10.1186/1532-429X-18-S1-P55

**Published:** 2016-01-27

**Authors:** Mohammed Shahid, Joseph Solomon, Francisco Contijoch, Brian Avants, Paul Yushkevich, James J Pilla, Yuchi Han, Walter R Witschey

**Affiliations:** 1 grid.25879.310000000419368972Radiology, University of Pennsylvania, Philadelphia, PA USA; 2 grid.25879.310000000419368972Medicine, University of Pennsylvania, Philadelphia, PA USA

## Background

Arrhythmias are a significant healthcare burden and an estimated 400,000 people die annually due to arrhythmia related complications. While pathological electrical activity can be observed at the endocardial surface during electrophysiology treatment, high spatial resolution non-invasive methods are needed to resolve aberrant contraction and establish correspondence with regional myocardial disease. To elucidate these abnormal electromechanical patterns, we sought to establish precise timing information of regional myocardial motion and global parameters using real-time MRI.

## Methods

This study was approved by the IRB at the University of Pennsylvania and all subjects gave written informed consent. 5 patients with PVCs and 3 control subjects with no history of CVD were recruited. CMR was performed on a 1.5 T MRI (Avanto, Siemens, Erlangen, Germany) using a 2D multislice, real-time, golden angle balanced steady-state free precession sequence with TE/TR = 1.4/2.8 ms, FOV = 220-300 mm, spatial resolution = 1.7-2.3 mm, bandwidth=898 Hz/pixel, slice thickness = 8 mm, total acquisition time = 16-22 seconds/slice to observe at least one PVC morphology. Images were reconstructed using sliding window, non-Cartesian SENSE with 34 views per frame (temporal footprint = 95 ms) and 3-lead ECG data was synchronized to each radial view. Patient beat morphology was segmented into 'interrupted', 'long', or 'regular' categories according to the observed RR interval. For each morphology, a single mid-ventricular short axis slice was divided into 6 circumferential segments and time-to-peak displacement (sec), velocity (mm/sec), radial and circumferential displacement (mm) and strain (%) and slice parameters (slice EDV, ESV, EF, SV, CO) were computed (CVI42, Calgary). Statistical measurements were 2-observer inter- and intra-observer repeatability, 2-way ANOVA and significance reported at p < 0.05.

## Results

There was good inter- and intra-observer agreement of time-to-peak displacement (r = 0.80, ICC = 0.75, p < 0.0001) and velocity (r = 0.75, ICC = 0.74, p < 0.0001), peak radial (r = 0.76, ICC = 0.74, p < 0.0001) and circumferential strain (r = 0.74, ICC = 0.73, p < 0.0001). Regional displacement time-to-peak was significantly increased for interrupted (t = 0.32 ± 0.05 sec) and regular (t=0.31 ± 0.02 sec) compared to post-PVC long RR (t = 0.24 ± 0.02 sec) and control (t=0.25 ± 0.01 sec) beat morphologies, however, peak velocity, radial strain and displacement was significantly reduced for post-PVC long RR beats (16.3 ± 1.5 mm/sec, 26 ± 4%, 3.9 ± 0.5) compared to controls (21.2 ± 1.8 mm/sec, 32.1 ± 4.5%, 5.5 ± 0.6).

## Conclusions

Precise regional motion information for arrhythmic beat morphology can be obtained and has good inter-and intra-observer repeatability. Significant differences were observed in regional beat timing and motion for different beat morphologies and compared to control subjects.

**Table 1:** Beat morphology hemodynamic parameters

	control	interrupted	long	regular/intermediate
EF (%)	48.1	52.1	38.1	48.3
sEDV (mL)	14.6 ± 2.7	18.5 ± 2.5	13.6 ± 2.4	21.5 ± 2.1
sESV (mL)	7.9 ± 3.3	9.3 ± 2.6	9.0 ± 2.9	11.5 ± 2.6
SV (mL)*	6.8 ± 1.5	9.2 ± 1.2	4.6 ± 1.3	10.0 ± 1.2
RR (sec)	0.96 ± 0.15	0.76 ± 0.12	1.2 ± 0.13	0.9 ± 0.12

* indicates p < 0.05, ANOVA

**Figure 1:**
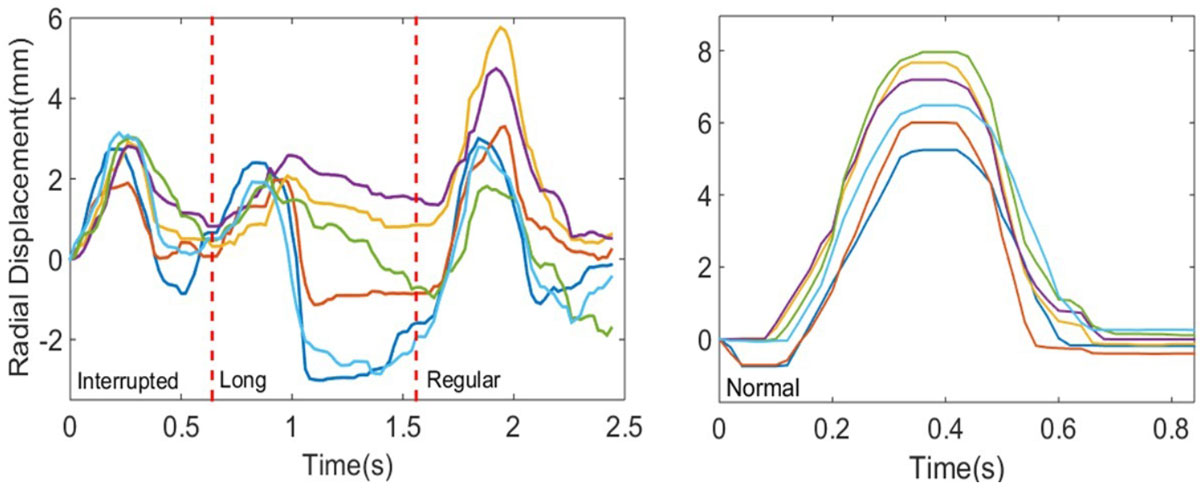
Figure 1

